# Gastrostomy as a Preemptive Measure after Pancreatoduodenectomy against Delayed Gastric Emptying: A Small Case Series and a Review of the Literature

**DOI:** 10.1155/2021/6649914

**Published:** 2021-02-23

**Authors:** G. Floros, I. Galanis, P. Theodoropoulos, D. Bartziotas, C. Theodoropoulos, M. Metaxa, G. Giannos, P. Tsintavis, G. Stylianidis, S. Klimopoulos

**Affiliations:** 2nd Surgical Department Evaggelismos Hospital, Athens, Greece

## Abstract

Delayed gastric emptying (DGE) is a common (20–30%) postoperative complication following pancreatoduodenectomy (PD) (Parmar et al., 2013). Various causes and preemptive measures have been suggested to decrease the occurrence of DGE. We added a simple step in the procedure of 26 consecutive pancreatic head resections, which seems to alleviate DGE and has never been highlighted before.

## 1. Background

Delayed gastric emptying (DGE) is a common postoperative complication following pancreatoduodenectomy (PD) that not only impedes the adjuvant therapy of the malnourished patient with pancreatic cancer but also increases the length of hospitalization.

The International Study Group of Pancreatic Surgery (ISGPS) defined DGE as the inability to apply a standard diet by the end of the first postoperative week. Categories of A, B, and C have been established considering the inability to tolerate a solid diet by postoperative days 7, 14, and 21 or requirement or reinsertion of a nasogastric tube after the 3^rd^, 7^th^, and 14^th^ postoperative days, respectively [1]. Various causes and possible triggers have been related to the occurrence of DGE [2–19]. These include acute changes in plasma gastrointestinal hormone (specifically motilin) levels due to duodenal resection [4–7], ischemia and congestion of the pylorus and antrum secondary to vascular compromise [9], and denervation of the stomach and duodenum due to radical resection of the surrounding tissue [8, 18] with subsequent pylorospasm [11]. Gastroparesis secondary to postoperative intra-abdominal complications is often, but not always associated with pancreatic fistula, peripancreatic fluid collections, or intra-abdominal abscess [2, 12, 13, 15, 16, 18]. Additional functional abnormalities include pancreatic fibrosis [17], preoperative cholangitis [14], postoperative pancreatitis [3], alternation of the endocrinologic milieu, and perioperative blood transfusion [18, 19]. Furthermore, the performances of classic Whipple procedure versus pylorus-preserving pancreatoduodenectomy (PPPD) [20], antecolic versus retrocolic gastric/duodenal reconstruction [21], hand-sewn versus stapled duodenojejunostomy, Billroth I versus II reconstruction, pancreaticogastrostomy versus pancreaticojejunostomy, and other operative factors that may impact the rate of DGE have been investigated [22–31]. Last but not least, technical errors such as torsion or angulation of the reconstructed alimentary tract causing DGE should be preventable [32, 33]. We added a simple step in the procedure of 26 consecutive pancreatic head resections, which seems to alleviate DGE and has never been highlighted before.

## 2. Patients and Methods

123 pancreatic head resections either with Traverso–Longmire (33) or with typical Kausch–Whipple (90) procedure were performed from 2012 to 2019 in the second surgical department of Evaggelismos general hospital. The selection of the procedure was based on whether the tumor invaded the duodenum or not. Mortality rate was about 16%. 26 patients received randomly a Witzel gastrostomy with gastropexy intraoperatively, which would be closed when the postoperative ileus subsided and the discharge from the gastrostomy tube was below 500 ml daily. Liquid diet (water, tea, and soup) would start on 5 pod and progressed depending on the patient tolerance. A gastrografin swallow was performed in addition to clinical assessment when the patients could not return to a standard diet by the end of the first postoperative week. If it would indicate slow gastric emptying, an opened pylorus, and an unobstructed antecolic gastrointestinal anastomosis, the gastrostomy tube was used as drainage. Upon tolerating solid diet, the patient would often be discharged with the gastrostomy tube closed and with instructions to appear after a week in our hospital to remove the tube.

## 3. Results

**Figure 1:**
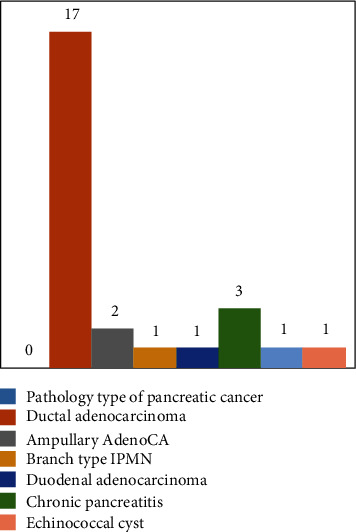
Pathologoanatomical studies of 26 PD.

Pathologoanatomical examination of the 26 (11 female and 15 male) specimens (Figure 1) after the operation demonstrated chronic pancreatitis in 3 patients, cancer of the ampulla of Vater in 4 patients, IPMN in 1, and cancer of the head of the pancreas in 17 patients. What should be highlighted is that the examination of all specimens revealed component of chronic pancreatitis and that perineural invasion was present in all specimens with pancreatic cancer. One female (30 years) had an echinococcal cyst in the head of the pancreas. 11 (5 female and 6 male) patients had a pylorus-preserving PD while the rest 15 (9 male and 6 female) a pylorus-resecting PD (Table 1).

Only four patients (15%), all of them with pylorus-resecting PD, exhibited signs of gastroparesis. One of them presented leakage from the pancreaticojejunal anastomosis, while another one presented postoperative hemorrhage. The rest twenty-two patients had no postoperative gastric atony, even though four of them presented anastomotic leakage. The mean RBC units perioperatively transfused were 3 per patient.

## 4. Discussion

Over time, it has been assumed that DGE is strongly associated with the occurrence of other postoperative complications such as fistula formation, postoperative sepsis, and reoperation. Whether the duodenum is preserved or not, DGE occurs after both classic (with antrectomy) and pylorus-preserving pancreatoduodenectomy [34–37].

Several therapeutic alternatives for the treatment of DGE are reported in the literature like the usage of prokinetic drugs (motilin, erythromycin, and cisapride), postoperative cyclic enteral feeding, and several specifications for reconstructing the alimentary tract, preservation of vagal pyloric branches, vascular structures, and length of the duodenum.

Since gastric atony after these procedures is common (20–30%), in anticipation for prolonged requirement of gastric drainage, we regularly use a Witzel gastrostomy.

Indications to perform a gastrostomy, in historical order of their development, include the following: (1) to provide nutrition, (2) to allow retrograde dilatation of the esophagus, and (3) to provide postoperative decompression of the gastrointestinal tract [38]. Gilchrist (1953) was an early advocate of using a gastrostomy tube to decompress the stomach postoperatively, to avoid the complications of an indwelling nasogastric tube and to promote patient comfort [39]. Warshaw and Torchiana [40] found that patients after PPPD frequently required prolonged nasogastric intubation and could not tolerate a regular diet in the early postoperative period due to DGE.

**Figure 2:**
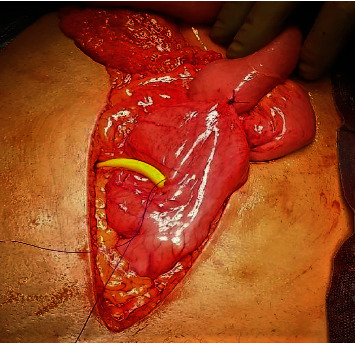
Two small holes, one in the center of the purse string suture (PDS 2-0) used to secure the tube after inserting it in the stomach and the other in the anterior abdominal wall, lateral to the left rectus muscle, are via electrodiathermy accomplished. A No. 18 Foley catheter is advanced through the anterior abdominal wall and into the body of the stomach, in a caudal direction. 3 ml of saline is used to inflate the Foley balloon so that it would not slip out of the stomach (we deflate it on the first postoperative day).

**Figure 3:**
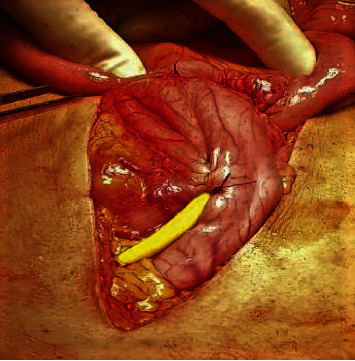
A serosa-lined tunnel (Witzel canal) is created with a continuous suture of the same 2-0 PDS used at the beginning.

**Figure 4:**
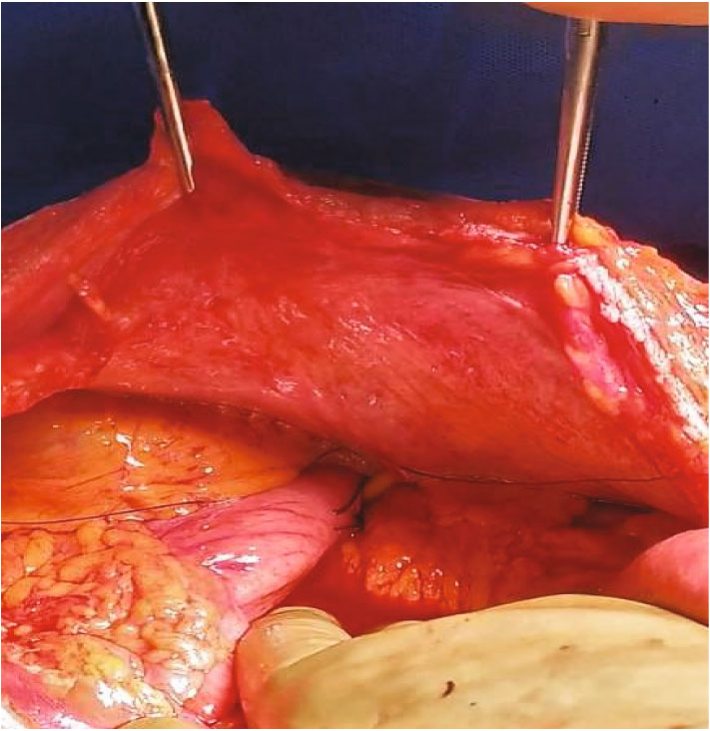
A gastropexy is performed with two single resorbable sutures, concealing the tube and anchoring the stomach to the parietal peritoneum of the left abdominal wall.

The Witzel technique [41], as described (Figures 2–4), has proved to be safe, reliable, and simple to perform. It offers the patient comfort (since a nasogastric tube is not necessary), immediate function for decompression, and low morbidity [40, 42]. Complications include internal migration, wound infection and peritonitis, when leakage occurs, or inability to remove it and dislodgement.

The site of insertion of the gastrostomy tube is selected along the greater curvature of the body of the stomach, within approximately 2 or 3 cm of the gastroepiploic branches and on the anterior wall of the stomach. The selection of this site is based on two speculations.

Firstly, fixating gastrostomy on the anterior and middle portion of the stomach might not affect both gastric fundic lag phase and gastric antral motility, which are considered important for gastric emptying [31].

Secondly, fixating the stomach to the anterior wall keeps it taut enough to prevent torsion or angulation of the reconstructed GI anastomosis. Combination of gastropexy with an antecolic anastomosis seems to facilitate passive emptying of food into the jejunum with the help of gravity.

The gastrostomy placement provides through its “gastropexy effect” a slightly increased low esophageal sphincter (LES) pressure [43]. The speculation that it may slow gastric motility and lead to slower function of the GI anastomosis after PPPD [44] was not confirmed in our study.

## 5. Conclusion

To date, there is no reference in literature about the use of gastrostomy to prevent DGE. Our small series of patients showed that gastrostomy-gastropexy can be used, with safety and efficacy, as a preventive measure for DGE in patients undergoing pancreatoduodenectomy. Further studies and randomized trials are necessary in order to establish safe conclusions.

## Figures and Tables

**Table 1:** The resected intestinal length in cm. The duodenum and jejunum are considered the main sites of motilin production. Conservation of the intestine as much as possible should result in more peristalsis and accelerated gastric emptying.

	Statistic
Mean	21,041
95% confidence interval for mean	
Lower bound	19,266
Upper bound	22,815
Median	22,000
Std. deviation	4,4852
Minimum	15,5
Maximum	32,0
Range	16,5

## Data Availability

The data that support the findings of this case report are available from the corresponding author, G. Floros, upon reasonable request.
